# Embedding patient safety in a scaffold of interprofessional education; a qualitative study with thematic analysis

**DOI:** 10.1186/s12909-023-04934-6

**Published:** 2023-12-18

**Authors:** Shaista Salman Guraya, Muhammad Umair Akhtar, Nabil Sulaiman, Leena R. David, Feras Jassim Jirjees, Manal Awad, Sausan AL Kawas, Mohamed Hassan Taha, Mohamed Haider, Jacqueline Maria Dias, Shada Aysha Kodumayil, Nihar Ranjan Dash, Amal Al-Qallaf, Ahmed Hasswan, Vida Abdolhamid Salmanpour, Salman Yousuf Guraya

**Affiliations:** 1 Royal College of Surgeons Ireland, Medical University of Bahrain, Busaiteen, Bahrain; 2 College of Medicine, University of Sharjah, Sharjah, United Arab Emirates; 3 College of Health Sciences, University of Sharjah, Sharjah, United Arab Emirates; 4 College of Pharmacy, University of Sharjah, Sharjah, United Arab Emirates; 5 College of Dental Medicine, University of Sharjah, Sharjah, United Arab Emirates

**Keywords:** Interprofessional education, Patient safety, Collaborative practice, Cognitive maturity, Socio-economic scaffolding

## Abstract

**Background:**

Regardless of a proliferation of interest in reducing unsafe practices in healthcare, threats to patient safety (PS) remain high. Moreover, little attention has been paid towards the role of interprofessional education (IPE) in enhancing PS. This qualitative study was conducted to unfold the insights of the senior medical, dental and health sciences students at the University of Sharjah (UoS) in the United Arab Emirates (UAE) about PS in an online IPE-based workshop.

**Methods:**

This inductive thematic analysis study was conducted on senior medical and health students at the Colleges of Medicine, Dental Medicine, Health Sciences, and Pharmacy of UoS. During an online workshop, students discussed plausible solutions for four real practice-based clinical scenarios with elements of unsafe healthcare practices. During the breakout rooms, the students exhibited high level of articulation and proactively participated in discussions. The data from the online workshop were transcribed and then coding, categorizing, and labelling of recurrent themes were carried out. Multiple individual deliberations, consolidation, incorporation of the identified preliminary themes, and merging and reorganizing sub-themes led to a final thematic framework.

**Results:**

This work delved into the perspectives of 248 students regarding teamwork, communication, problem-solving, and other aspects concerning PS in interprofessional settings in an online workshop. The iterative process of data transcription, curating and qualitative analysis surfaced 32 codes. Later, the inductive themaric analysis yielded five themes with distinct yet interconnected nested subthemes in the context of PS in IPE settings. These themes of information sharing and grounding (problem-solving, social skills), maintaining communication (clinical reasoning, shared mental model), executing interprofessional activities (collaborative practice, collaboration scripts), professional cognitive abilities (cognitive maturity, metacognition), and negotiating professional identities (systematic change, socio-economic scaffolding) emerged as fundamental pillars for enhancing PS in healthcare.

**Conclusion:**

Our study demonstrated the outcome of an innovative and team-based workshop which embedded PS within a scaffold of IPE environment. This research calls for incorporation of the emerging areas of clinical reasoning, problem solving, collaborative practice, and shared mental model into medical curricula for structured IPE in improving PS domains in medical education. These findings underscore the need for multifaceted dimensions of IPE imperatives for cultivating collaborative competence.

**Supplementary Information:**

The online version contains supplementary material available at 10.1186/s12909-023-04934-6.

## Background

According to the World Health Organization (WHO), patient safety (PS) focuses on preventing medical errors and their unfavorable effects on patients during the delivery of healthcare systems [1, 2]. An unsafe health care practice potentially leads to harmful damage to patients in the form of injury, death, or disability [3]. There is a staggering rise in the occurrence of unsafe medical practices worldwide. As reported by WHO, one in 10 patients is subject to unsafe medical events during hospital care and such compromised delivery of health care leads to 134 million adverse outcomes with an estimated 2.6 million deaths annually [4]. In order to eliminate avoidable harm to patients, the United Nations has introduced sustainable development goals with the Global Patient Safety action plan 2021–2030 [5].

PS is a fundamental component of strategic targets of healthcare systems worldwide [6]. Additionally, PS is a complex and multifaceted responsibility which is influenced by a wealth of factors, including interprofessional education (IPE), interprofessional collaboration (IPC), effective communication, and professional behaviors [7–9]. IPC is understood as a phenomenon where healthcare professionals from different healthcare disciplines work with patients and their families to enhance the quality of healthcare [10], whereas IPE are occasions where students from different healthcare disciplines learn with, from, and about each other [11]. Both IPE and IPC promote PS as healthcare professionals (HPCs) work together and negotiate their professional identities and roles while managing patients in healthcare facilities. At the same time, IPE and IPC are considered the major determinants of safe healthcare practices, enhancing effective communication, shared decision-making, and teamwork [12]. Several studies have reported that IPE and IPC among students and HCPs, respectively, improve understanding about PS and lead to better healthcare outcomes [13, 14].

Despite their critical roles in healthcare systems, IPE and IPC have not gained enough popularity worldwide. Reluctance to collaborate, lack of understanding of professional roles and responsibilities, working in silos, and an absence of teamwork among HCPs are some deterrents towards IPC. Ironically, the root of these limiting factors of IPC can be traced back to a glaring omission of IPE-based curricula in most medical institutions [15].

Despite a proliferation of interest to improve PS and to reduce errors in health care by training undergraduate medical and health sciences students in a climate of IPE, there is a scarcity of evidence toward a coherent effort in educating future healthcare professionals. This highlights the need for an intensified effort towards training the medical and health sciences students in an-IPE based environment. Our qualitative interventional research envisaged to determine the understanding and insights of senior medical, dental and health sciences students at the University of Sharjah (UoS) in the United Arab Emirates (UAE) about PS in a virtual climate of IPE-based workshop. Additionally, the study evaluated the feasibility of this interventional workshop in fostering the understanding of the same cohort of students. The findings of this qualitative analysis can potentially help medical and health sciences educators to inculcate IPE in curricula using the identified themes and relevant concepts.

## Materials and methods

### Study objective

This qualitative study aimed to probe the understanding and insights of senior medical, dental and health sciences students at UoS in the UAE about PS in an IPE-based educational workshop. At the same time, our research explored the feasibility of the IPE-based workshop in fostering the understanding of the participants about PS. The workshop was conceived with an idea to analyze real-life incidents with threats to PS and how IPE could have prevented or mitigated such errors or near-misses. The workshop aimed to explore ways to promote a PS culture within inter-professional teams by emphasizing, reporting, and learning from errors.

### Study design

We chose a qualitative study design as this type of research is a well-established and widely used approach for investigating complex phenomena of healthcare education on PS domains [16]. In particular, qualitative inquiry is well-suited to understand interactions among participants and healthcare settings as this strategy permits an in-depth exploration of participants’ subjective views and experiences and the meanings they attach to them [17]. This mechanism resonates well with the interpretivist approach, which focuses on understanding the phenomenon from the perspective of individuals [18]. We adopted the inter interpretivist paradigm approach which can be described as ontological, relativism, and epistemological subjectivism. Ontological relativism acknowledges the subjective nature of reality, recognizing that reality is individually constructed [19]. On the other hand, epistemological subjectivism focuses on the understanding that knowledge is subjective and is influenced by individual perspectives and interpretations, though grounded in real-world phenomena [18].

### Clinical cases with potential harm to patient safety used in the online workshop

For the small group interactive discussions of students in the online workshop, we prepared four clinical cases with potential threats to patient safety. These practice-based cases were witnessed and reported by students of the CoM at UoS during their training in clinical clerkships. The research team chose these clinical cases with elements of; (a) diverse representation of all medical and health disciplines, (b) apparent compromise or threat to PS, and (c) appropriateness of the clinical area for senior undergraduate students. We adopted the Biggs’ 3P’s approach to learning and assessment for our study [20]. The 3P’s - *Presage*, *Process*, and *Product* - encompass factors influencing learning processes and outcomes [21]. In our study, the *presage* refers to the initial selection of cases, while the *process* involves a detailed analysis and discussion conducted during the breakout sessions. The resulting *product* of this study encompasses the insights, recommendations, and broader implications for PS in undergraduate education. A description of each case is available in Appendix I.

An overview of key features of each clinical case is summed up in Table 1.

**Table 1:** Overview of four clinical cases discussed in the supervised interactive small group discussions during the online workshop

	Case description	Patient safety domains
**Case 1**	Unrecorded insulin dose led to overdose.	Professionalism
**Case 2**	Lack of instructions caused opioid overdose.	Professionalism, communication
**Case 3**	Infection risk from shared bed linen.	Professionalism, leadership
**Case 4**	Wrong tooth extracted during oral surgery.	Professionalism, leadership

### Workshop participants

The study was conducted on the currently enrolled senior students at the College of Medicine (CoM), College of Dental Medicine (CDM), College of Health Sciences (CHS), and College of Pharmacy (CoP) of the UoS. At the time of the IPE-based workshop, the students were studying in the final two years of their respective programs. The four medical and health colleges at UoS primarily use problem-based learning curricula and, per se, do not have an accredited IPE-based course or program. However, PS is focused in all educational and training pedagogies across the medical campus of UoS. Recently, the committee for interprofessional education and practice in the medical campus of UoS has significantly transformed the culture and attitudes of faculty and students towards IPE and collaborative education. The facilitators of the online workshop included the faculty and clinical tutors from UoS and the Medical University of the Royal College of Surgeons (MUB-RCSI) Bahrain.

For our research-based online workshop, invitations containing informational flyers, registration links with QR codes, and written consent forms were sent to all eligible students via email. A participant information leaflet was provided to all registered students for their prior understanding and orientation about the workshop.

### Workshop structure

**Fig. 1:**
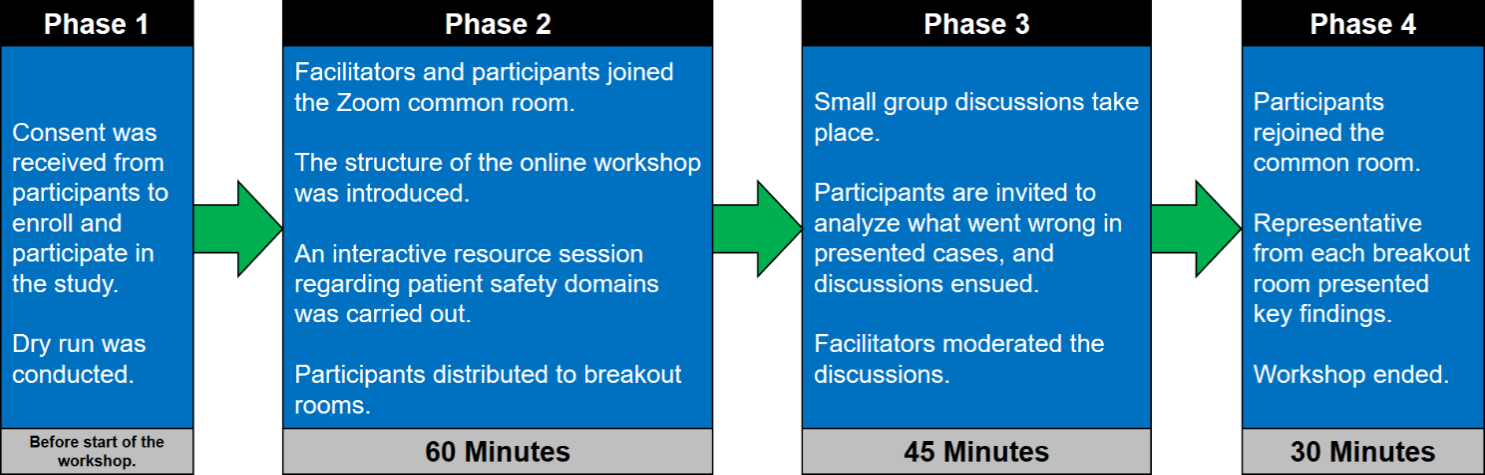
A chronological order of the phases of the research with details of the online workshop on the medical and health colleges students at the University of Sharjah

The online workshop was conducted on the Zoom application. The workshop was carried out in four distinct phases **(**Fig. 1**).**

Phase 1 included pre-workshop planning and preparations, securing written consent from participants, conducting a dry run to ensure online training of facilitators and organizers, internet security, breakout rooms, power point presentations, and equipment. In phase 2, during the online workshop, all participants and facilitators joined the Zoom main meeting room, where researchers delivered three interactive resource sessions which primarily focused on patient safety, medical professionalism, leadership in healthcare, and the interconnectedness of these domains in improving healthcare outcomes for PS. This phase lasted for 60 min.

In phase 3, participants were divided into 20 breakout rooms to engage them in IPE-based small group discussions. The composition of students and facilitators in each breakout room was carefully planned to ensure equal representation from each college and discipline, fostering an authentic IPE environment for both students and facilitators. By utilizing such small group interactive discussions, we created a collaborative and participatory opportunity where students could express their thoughts freely about PS domains collectively in an interprofessional setting.

Within breakout rooms, lasting for 45 min, participants were presented with four clinical cases that posed various threats to PS. These cases were read aloud by a group leader in each breakout room, appointed by the facilitator. The students were encouraged to actively engage in the discussions using the Borton’s reflective process approach, which was also adopted into Rolfe et al.’s reflexive model [22]. This approach is based upon three simple questions: ‘what?’, ‘so what?’, and ‘now what?’. Facilitators provided guidance and assistance throughout small group discussions, fostering a collaborative and interactive environment. The main sequence of events included identifying incidents of malpractice in each case, recognizing potential underlying causes, selecting patient safety domains, analyzing possible measures to prevent similar incidents, and reflecting on how IPE and IPC could contribute to PS.

In phase 4, after the breakout sessions, all participants reconvened to the main workshop room, where a student spokesperson from each breakout room group presented a summary of their small group discussions and proposed solutions to clinical scenarios. The workshop concluded with a wrap-up session, expressing gratitude to the organizers, students, facilitators, and IT support staff involved in the event. This final phase lasted for 30 min.

### Data collection

We collected data of recordings of all parts of the workshop, including small group and common room discussions using the Zoom’s built-in meeting recording feature. Following the workshop, the recordings were transcribed verbatim using the speech transcribing software, Trint and Microsoft Word. Selected transcriptions were then meticulously proofread to minimize potential errors and to enhance the data reliability. The utilization of recordings and transcription facilitated a detailed analysis of discussions and enabled the researchers to revisit and delve deeper into the participants’ perspectives and opinions.

### Data analysis

Thematic analysis is a methodological framework that involves identifying, analyzing, and interpreting patterns within qualitative data to comprehensively understand the research topic [23]. Through a rigorous process of coding, categorizing, and labelling recurrent themes, we aimed to uncover the students’ insights of underlying factors, perspectives, and challenges related to PS in the context of IPE. We adopted the Braun and Clarke’s 6-step thematic analysis approach which includes; become familiar with the data, generate initial codes, search for sub-themes and themes, review themes, refine themes, and write-up [24]. By employing thematic analysis, we sought valuable insights into the students’ experiences and certain areas of improvement regarding PS in an IPE environment. In our research the inductive thematic analysis was adopted for qualitative analysis which is an established form of qualitative inquiry, well-suited for healthcare research [25]. In the inductive thematic analysis, themes are built up from the data itself, and transcripts are not read with preconceived notions or predetermined data points. Rather, transcripts are analyzed for latent meanings and coded accordingly [25].

**Fig. 2:**
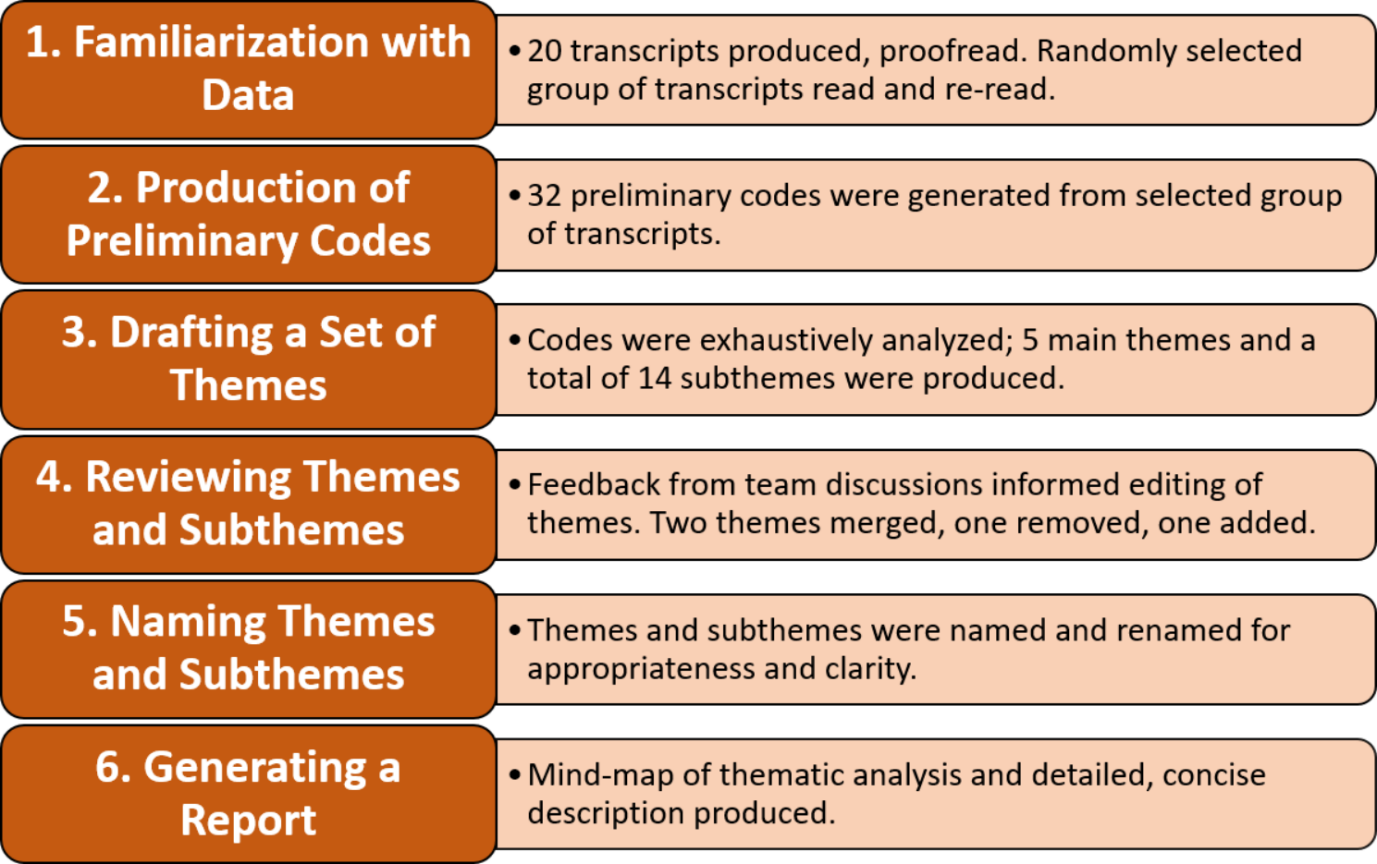
A stepwise data analysis of the qualitative data as developed by Braun and Clark’s 6-step approach

In the first step, MUA, SSG and SAK read and re-read a randomly chosen a set of three transcripts to familiarize themselves with the data and noted down main points for analysis. Afterwards, 32 codes were extracted from the transcribed data. A shared understanding of the meaning of the identified codes was established among research team members and these were then used to draft a preliminary set of five themes. Feedback from team discussions was instrumental in refining the process of thematic analysis. During the first two of three rounds of discussions, a conceptual thematic framework comprising od sub-themes and themes was identified and agreed upon. However, re-reading the transcript sets, multiple individual deliberations, consolidation, and integration of identified preliminary themes, and merging and reorganizing sub-themes led to a final thematic framework. Through this iterative process, the research team included all other researchers and achieved a consensus on the last set of themes and subthemes, ensuring the accuracy and comprehensiveness of the analysis. As a final step, a mind map of the selected themes was produced, and the results section of this paper was penned down, with a detailed elaboration of data analysis **(**Fig. 2**).**

## Results

There were 248 students from four medical and health colleges of UoS. There were 118 (47.6%) students from CoM, 91 (36.7%) from CoP, 33 (13.3%) from CHS, and 6 (2.4%) from CDM.

**Fig. 3:**
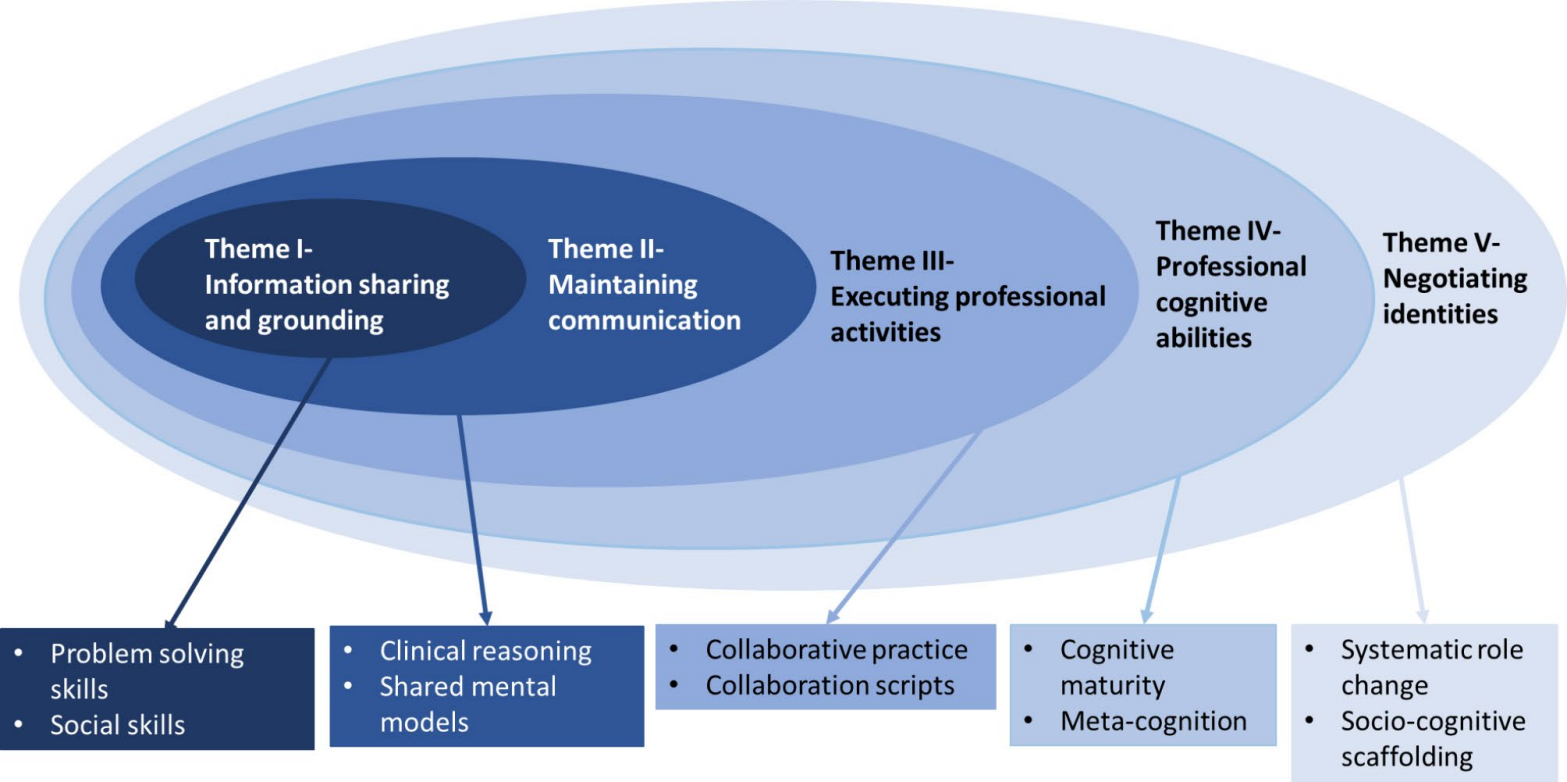
Themes, sub-themes, and their inter-connectedness in the form of an onion ring relationship, as generated during the thematic analysis

The workshop sheds light on the promotion of the culture of PS using IPE as a vehicle for team-working and shard decision making. The thematic analysis revealed 10 subthemes and five prominent themes which were generated from 32 codes **(**Fig. 3**)**. As illustrated in the onion ring diagram, the inductive qualitative analysis yielded five interrelated themes where a nested relationship seemed the most appropriate, keeping the context of interprofessional collaborative practice in healthcare settings.

The first theme, ‘information sharing and grounding,’ represented the seed of the nested ‘matryoshka dolls’, and the final and fifth theme of ‘negotiating identities’ surfaced as the matriarch of the analysis. This relationship highlights the significance of all identified themes. The first theme, “information sharing and grounding,” emphasizes the significance of effective information exchange within healthcare teams. It highlights the challenges related to information sharing in problem-solving situations among professionals. The second main theme “maintaining communication,” underscores the importance of advocating clinical reasoning in each step taken by healthcare professionals and ensuring the existence of a shared mental model in patient care. The third theme, “executing interprofessional activities,” builds on the first two themes and focuses on effective collaboration and coordination among healthcare professionals and strategies. Furthermore, the fourth theme, “cognitive professional abilities,” interconnect individual cognitive skills and abilities that impact students’ understanding of PS outcomes such as maturity and cognitive power. The final theme, “negotiating professional identities,” delves into the challenges and dynamics of professional roles within healthcare teams. Identities become malleable once all the preceding attributes are well nurtured in various members of healthcare teams. It explores aspects such as the distribution of power and responsibilities within teams, and the collaborative support and learning provided by team members.

These themes provide valuable insights into the complex interplay of various factors influencing PS in an interprofessional context, informing the development of targeted interventions and strategies to enhance healthcare outcomes in healthcare. A brief account of the analysis of each theme alongside its relevance to our research, including specific excerpts, is provided hereunder.

### Theme I: information sharing and grounding

Information sharing and grounding are two crucial concepts to ensure a culture of patient safety by incorporating *problem solving* and *social skills*. This encompasses sharing relevant patient details, investigation results, management plans and any critical information using standardized protocols among various healthcare team members.

#### Problem solving abilities

Participants recognized that inadequate conveyance of information was a fundamental cause of the underlying numerous threats to PS presented during the small group discussions. Specifically, a notable subtheme that surfaced from the participants’ discussions highlighted the correlation between deficient information sharing and a subsequent dearth of problem-solving capabilities within healthcare teams.

If there was good communication between the first and the second nurse, this would have been avoided. That’s an easily avoidable problem.

#### Social skills

Additionally, participants demonstrated an understanding that deficient social skills related to sharing ideas posed a significant barrier to the effective exchange and grounding of information. They acknowledged that the ability to convey ideas and insights efficiently is crucial for effective information sharing.

*“I think it would be better that we make sure doctors and nurses, for example, in this case, have a better working chemistry and that there are no barriers in communication. Even if the nurse wants to say something which she would be otherwise uncomfortable to share with the doctor if she’s not like, as I said, not in coherence with the doctor.”*

Despite a collegial and seamless continuation of discussions, there were occasions where participants were not able to interact with each other to discuss solutions extensively.

*“Could you just tell me what patient safety domain is highlighted here?*

Doctor, is there a communication error?

Mostly, facilitators shared some triggers at nodal points to facilitate deliberations and to keep a set pace and direction of small group sessions.

### Theme II: maintaining communication

In healthcare context, *shared mental models* and *clinical reasoning skills* are inevitable to maintain communication, leading to successful patient outcomes and effective teamwork.

#### Shared mental models

By promoting clear and open communication, healthcare professionals can foster a shared understanding and enhance clinical reasoning, leading to improved clinical outcomes and a safer healthcare environment [26–29]. Shared mental models are intricate cognitive frameworks that are individually held by healthcare professionals and serve to facilitate collaborative functioning within clinical settings. These cognitive structures enable healthcare teams to navigate complex healthcare environments effectively by promoting a collective understanding of the team’s tasks, goals, strategies, and unique roles [28, 30]. As healthcare necessitates precise and coordinated actions, cultivating shared mental models is essential in optimizing patient care outcomes and enhancing interdisciplinary teamwork [30, 31]. An interesting excerpt in this context is reported by one of the participant is

Again, the implementation of communication and maybe the nurse have also to recheck if maybe the general examiner or anybody before her just sanitized or changed the sheets or not. So as we can say, it’s a shared responsibility.

One observation made throughout this study was that students from the same school showcased a similar level of understanding and knowledge and were more likely to build a rapport with each other. However, students contributed more actively in the case/s where the patient’s main health problem was related to their specific subject.

The patient safety issue is procedural and surgical errors, while the patient safety domain is professionalism and leadership. The lack of proper communication, leadership, and decision-making led to misidentification and wrong diagnosis and management.

#### Clinical reasoning skills

Effective communication is integral for clinical reasoning involving critical questioning, interdisciplinary collaboration, and patient involvement [32]. Participants were cognizant of all these aspects; however, prompts by the facilitators were needed to improve the interprofessional discussions. Expert facilitation was needed to work through various layers of problems which were presented and solved systematically. Some interesting triggers and cues are reported here.

What should be done to prevent such mistakes in the future?

*“Double-check and making sure you have the right placement.*

How can that be done?

Always suspect, always double-check and listen. Proper communication with the patients and active listening.

### Theme III: executing interprofessional activities

In healthcare, most educational activities aim to foster a patient–centric culture. Future healthcare professionals should be aware of potential *collaborative scripts* encountered in the clinical practice which will help them to implement and sustain *collaborative practice*.

#### Collaborative practice

During the workshop, participants were able to understand and explain the importance of IPE accurately and they recognized that effective teamwork and collaboration among different healthcare professionals remains vital for ensuring PS.

Opioids are high alert medications. There’s no way you can just administer an opioid like with one order. When you administer an opioid, there’s always two nurses and then the in-charge himself to sign that as well. …….Why are you all signing off on it?

I think in a busy clinic, they should, like, manage their time and be more organized between, like, each patient and communication with each other between each patient to tell if they’re doing their job right or not.

#### Collaborative scripts

Furthermore, some participants exhibited a tendency to focus solely on specific reasons for errors in healthcare without a comprehensive understanding of standardized safety protocols. However, their peers were able to make them understand the difficult concepts during the small group discussions. This underscores the significance of IPE based collaborative scripts in teams in healthcare education.

Speaker 12: Doctor, we also can include the pharmacy if they put like a red flag that two doses of insulin were requested for the same patient.

Something like that can be done in the system itself. The EMR can have an application for something like that.

### Theme IV: professional cognitive abilities

Combined, *cognitive maturity* and *meta-cognition skills* enhance professional competence and optimize patient care [32].

#### Cognitive maturity

Critical thinking and problem-solving skills add to the cognitive domain and lead to the identification of systemic inefficiencies which, in turn, would potentially result in effective solutions.

I wanted to ask whose responsibility it is to provide instructions to the patients for getting into the MRI?

However, a spectrum of cognitive maturity was evident from the participants’ discussions in interprofessional climate. Differences in cognitive maturity between medical and health colleges students were evident, with medical students being more well-articulated, showing their cognitive maturity. Prompt initiation of discussions during relevant cases by medical students depicted an efficient approach to acquiring, processing and utilization of information.

Could you just tell me what patient safety domain is highlighted here?

Doctor is there a communication error?

What should be done to prevent such mistakes in the future?

#### Metacognition

While metacognition is an introspective process to enhance one’s cognitive abilities, self-reflection, awareness of cognitive bias and monitoring lead to improved decision-making. Metacognitive abilities facilitate healthcare professionals to act soundly in high-pressure situations. There were some glimpses of this desired attribute, though we expected more.

Before undergoing any sort of procedure, make sure that every point of contact with any health care professional, that the procedure is explained again and again.

During the small group discussions, it was evident that medical students had a greater understanding of protocols such as hand hygiene and were able to discuss proactively. However, all students were exhibited some level of articulation and incorporated appropriate jargons and medical terminology within their answers.

The nurse is wrong, she forgot to write or document the problem. The dose, I think.

We can see that she forgot to record the subcutaneous dose on the patient file.

### Theme V: negotiating professional identities

Identity negotiation is a process that involves recognizing and acknowledging individual and societal perceptions of reality while also confronting and questioning one’s own identity and the identities of others within diverse cultural contexts [33]. In healthcare, negotiating identities involves *systematic role change* and its *socio-cognitive scaffolding*. These two essential concepts play a significant role in the execution of cohesive teamwork.

#### Systematic role change

By breaking down the silos, healthcare professionals periodically take on the responsibilities of their colleagues from other disciplines. This flexibility and adaptability are a positive add-on to the complex, demanding patient care culture.

So we’re talking about something that has different people involved. We just mentioned nurses, pharmacists and doctors. So this is interprofessional. And if there was better communication between the three fields or the three different—staff, I think that’s the word—someone would have noticed something is wrong. Like, it’s not the sole responsibility of the nurse. It’s not the sole responsibility of the doctor, and it’s not the sole responsibility of the pharmacy either. It’s something collective.

Doctor, speaking from my experience, when I’m doing my rotations in the OPD, I always make sure that the bed linen’s changed. Also, most of the times, I change it myself because I know that nurses are busy outside and doctors are also busy using his computer. And so I think medical students can also step ahead and do this little task for the doctor or the nurse because it’s not a big of a deal anyways.

#### Socio-cognitive scaffolding

On the other hand, most of the answers by students were brought about using socio-cognitive scaffolding. It was heartening to see the socio-cognitive scaffolding of the systematic role change in our future healthcare professionals, who articulated an understanding of a shared mental model. This understanding is crucial among healthcare professionals to promote effective teamwork and a collaborative approach to patient care.

I think it’s a shared responsibility, but I think it’s also shared leadership because the nurse is the one administering the medication. So when the doctor orders a medication, it’s the nurse’s job to make sure that this medication hasn’t been administered or if it’s been administered. So even the leadership, it’s shared, it’s interprofessional. That’s what the point of interprofessional practice.

However, not all participants were able to depict this socio-cognitive maturity. Some responses indicated the blame and shame culture prevalent in the complex healthcare set-up representing the proximal participation in their communities of practices. Participants usually put the main responsibility of PS on medical professionals, highlighting the power hierarchy of healthcare culture.

I feel that the nurse or the GP should have been taking care of the entire thing, not instructing the mother instead….

## Discussion

Our study showed the current understanding and attitudes of medical, pharmacy, dental and health sciences students about PS in an interprofessional climate. The inductive thematic analysis of discussions about the commonly encountered clinical scenarios in healthcare settings depicting less optimal patient care yielded a spectrum of understanding and insights. The participants could identify a glaring absence of guidelines which could help them solve clinical scenarios even in multi-disciplinary teams. This study emphasizes the significant role of IPE-based teamwork among medical and health students using evidence-sharing and collaborative problem-solving skills to enhance PS. Our analysis demonstrated a correlation between five interconnected but still distinct themes of interprofessional collaborative practice in healthcare which can result in optimal outcomes related to PS.

The first two themes, ‘information sharing and grounding’ and ‘maintaining communication’ sit at the heart of the nested hierarchy of our thematic analysis. Published literature has emphasized communication as a distinct core competency for collaborative practice in care provision settings [34–37]. However, the discipline-specific language impedes communication, highlighting the importance of shared mental models and use of common language. This awareness of differences in professionals’ language demands an introspection to one’s social, problem solving and clinical reasoning skills. All accreditation and licensing bodies endorse the ideal portrayal of a healthcare professional who can pass on information and hand over care in the interest of patients while demonstrating flexibility, adaptability and a problem-solving approach [34]. In our work, participants from various disciplines identified the importance of ‘chemistry’ between colleagues, leading to shared mental models about ‘prioritizing the patient’. However, we noticed a significant lack in strategizing effective coordination due to a lack of objectivity and emotional maturity. This varying level of participation points towards the importance and presence of communication skills teaching in the curricula of various disciplines [38]. To ensure the success of this important concept of PS, ongoing efforts are needed to enhance the communication skills of individuals who have not had comparable foundation training in their education [39]. Faculty members need to carefully assess the pros and cons and expand their efforts into real-world applications to bridge the divide between academic learning and practical implementation [40].

In our study, third theme, ‘executing interprofessional activities’, was generated from two sub-themes – collaborative practice and collaborative scripts. Most students had a clear understanding of the importance of practice in collaborative settings but lacked an intimate familiarity with the unfolding of these collaborative scripts. This finding highlights the sparsity of collaborative educational activities throughout the curriculum. A prompt integration of IPE-based courses among medical and health sciences students will potentially equip them to navigate interpersonal dynamics. Collaborative scripts are predefined sequences of actions and dialogues whose curricular incorporation can enhance standardized and coordinated interactions among learners, upskilling their technical skills and interpersonal aptitudes [41]. Students spending time together in structured and collaborative activities would build confidence in comparable situations in practice, ensuring optimal patient outcomes [42]. By immersing students in simulated scenarios reflective of real-world healthcare contexts, IPE settings facilitate the development of teamwork, empathy, and shared decision-making by reducing the germane cognitive load inherent in the clinical learning environment [43, 44].

The fourth theme of ‘professional cognitive abilities’ surfaced from the interplay between the professional cognitive abilities and their impact within an interprofessional setting. Individual professional competence in terms of interprofessional capabilities necessitates a broader interpretation of metacognition. Educators need to integrate metacognitive theories into the realm of IPE to guide interprofessional team members in contemplating metacognitive procedures [45, 46]. This entails interventions to effectively manage interdisciplinary team collective reservoirs of knowledge and skills for the purposeful pursuit of patient safety culture. Recent research in higher education reported a positive impact of collaborative scripts employed to facilitate shared metacognitive regulation of participants engaged in interactions related to team planning and the construction of knowledge [46]. Essentially, the convergence of collaborative practices and scripts can redefine how professional activities can be executed within the patient-centric transcending disciplinary confines [47, 48]. Reflective practice can prepare the next wave of healthcare professionals to prioritize patient safety and excel in interprofessional collaborations by using a spectrum of *‘in action’* to *‘on action’* and *‘ for action’* reflective practices enhancing collaborative competence [49].

Our last theme of ‘negotiating professional identities’ emerged as a systematic role change of interprofessional team members and their socio-cognitive scaffolding of these changing roles. The fundamental objective of health professions education is to support and develop the professional identity of a learner in the communities of practice. A healthcare professional works in multiple communities of practices and manages and adapts by negotiating identities in multiple ways. Longitudinal integrated clinical placements from early educational journeys help scaffold the “being interprofessional” to the *self* of the professional [50, 51]. Such placements can lead to an unconscious comparison between one’s and others’ regions and terrains, resulting in nurturing and developing malleable professional identities fit for patient-centric practices [52, 53].

### Study limitations

Although each college of UoS had representation of students in the workshop, CDM had the least number of contributions. This might have skewed the results, but due to the qualitative nature of our study, this factor will have minimal impact on the outcome.

## Conclusion

Our study reports a successful outcome of an innovative multi-disciplinary, IPE-based online workshop with resultant thematic analysis, which emerged from deliberations of undergraduate medical and health sciences students about PS. These themes of information sharing and grounding, maintaining communication, executing interprofessional activities, professional cognitive abilities and negotiating professional identities showcase an interconnected foundation for both IPE and PS. Designing a curriculum using these domains will enhance soft skills of future doctors such as collegial learning, clinical reasoning and team-working using cognitive maturity and collaborative practice. This learning pathway will eventually enhance PS with better healthcare outcomes in the long run. The workshop enforces the role of IPE in promoting the collaborative role of IPE among medical and health sciences students about PS.

### Electronic supplementary material

Below is the link to the electronic supplementary material.

Supplementary Material 1

## Data Availability

The raw dataset and other materials are available on request. The corresponding author will provide additional data if requested.
