# Supplementary material for: Embedding patient safety in a scaffold of interprofessional education; a qualitative study with thematic analysis
Source: BMC Med Educ. 2023 Dec 18;23:968. doi: 10.1186/s12909-023-04934-6 (PMC10729414; doi:10.1186/s12909-023-04934-6)
Supplement: Supplementary file 1 — Supplementary Material 1 [file 12909_2023_4934_MOESM1_ESM.docx]

**Appendix 1**

**CASE 1:**

A 45-year-old man with Type I diabetes mellitus was admitted in the Emergency Department (ED) with complaints of lethargy, decreased oral intake and blood sugar levels of 850mg/dl. A diagnosis of Diabetic Ketoacidosis (DKA) was established.

After necessary measures, the blood parameters and the patient's lethargy improved. The attending physician ordered the resident to switch IV insulin to Subcutaneous (SC) long-acting insulin. Duty Registered Nurse (RN) administered the SC dose before going on a break but forgot to record it in the patient file.

Half an hour later, upon checking the patient's status, the resident instructed for a stat switch of insulin. The covering RN requested the pharmacy for the SC dose and administered it as per the resident's directions.

An hour later, the patient became drowsy and lethargic again. Blood tests revealed a sugar level of 55mg/dl.

- **Case 1 patient safety domain**: professionalism (lack of interprofessional communication and team work leading to medication error).

**CASE 2:**

At a tertiary care hospital, a restless elderly man with excruciating back pain was scheduled for a spine MRI to rule out a back abscess.

As the patient needed to remain still during the MRI time, the attending physician ordered a STAT dose of Opioid for the patient, which was given to the patient to reduce his restlessness.

The patient was not given any instructions before starting the MRI. During the MRI, he remained restless and was moving a lot, which resulted in a poor quality MRI image. The radiographer was pissed off and complained to the physician about the poor quality of the MRI image obtained and the need for repeating the MRI again. Consequently, the physician ordered for two additional doses of sedatives, attempting to stop the patient’s restlessness before repeating the MRI again.

Soon after receiving the third dose, the elderly man became less responsive and hypotensive which ultimately led to emergency airway intubation due to respiratory depression.

Later on, an inquiry was set up whose report revealed that the respiratory depression was attributed to receiving the sedative medications.

- **Case 2 patient safety domain:** professionalism (lack of interprofessional communication and team work / mismanagement).

**CASE 3:**

A 45-year-old female presented to a busy outpatient clinic with complaints of fever, cough, and runny nose for the past three days.

The General Practitioner (GP) examined her on the bed while she was having bouts of coughing. She was diagnosed with acute viral nasopharyngitis and discharged on supportive medications.

The following patient, a 2-month-old baby, presented for vaccinations and a well-baby check-up. The GP instructed the mother to put the baby on the bed, while the nurse had not sanitized and changed the bed linen.

- **Case 3 patient safety domain:** leadership + professionalism (lack of proper interprofessional communication and team work).

**CASE 4:**

A Dentist recommended that, as part of his treatment plan, a child’s upper first pre-molar tooth should be removed by an oral surgeon, and referred the patient to him accordingly.

Upon visiting the oral surgeon, patient’s mother insisted on being there during the extraction procedure, and indicated that the lower first pre-molar tooth has to be removed. The oral surgeon did NOT check, but extracted the tooth and the child left the surgery.

A few minutes later, the receptionist told the oral surgeon that the mother said the wrong tooth had been removed. The referral letter showed the mistake.

Now, the oral surgeon has been served with a legal notice.

- **Case 4 patient safety domain:** professionalism + leadership (lack of proper communication, leadership & decision making, which led to misidentification, wrong diagnosis & treatment).

**4 Discussion Questions:**

1. What went wrong?
2. What patient safety domain is highlighted here?
3. What should be done to prevent such mistake(s) in the future?
4. How does interprofessional practice contribute to patient safety in this case?
